# Neonatal Achalasia Cardia: A Case Report

**DOI:** 10.7759/cureus.67954

**Published:** 2024-08-27

**Authors:** Srilaxmi Nelakurthi, Vijayalakshmi Bheemireddy

**Affiliations:** 1 Department of Pediatrics, NRI Medical College & General Hospital, Guntur, IND

**Keywords:** heller’s esophagomyotomy, esophageal manometry, barium swallow study, failure to thrive, aspiration pneumonia, motility disorder, neonate, achalasia cardia

## Abstract

Achalasia cardia is more common in adults between the ages of 30 and 60 years. It is relatively uncommon in children and very rare in infants. Only a few cases of infants with achalasia have been reported till now. It is a motility disorder of the esophagus due to a failure to relax the lower esophageal sphincter. The common clinical presentations in infants are regurgitation, vomiting, respiratory symptoms, and failure to thrive. This can be easily misdiagnosed as gastroesophageal reflux disease. Surgical management is the mainstay of treatment. Here, we present the case of a female newborn with symptoms suggestive of achalasia from day one of life and successfully treated with Heller’s esophagocardiomyotomy and Nissen’s fundoplication, following which the baby is asymptomatic and thriving well.

## Introduction

Achalasia can present at any age and is best described as a readily treatable esophageal motility disorder. The pathophysiology behind achalasia is the degeneration of myenteric neurons that innervate the lower esophageal sphincter (LES) and esophageal body. There is an imbalance between excitatory and inhibitory elements of the enteric nervous system [1]. The incidence of achalasia is 0.11/100,000 children per year. The first case of achalasia cardia was reported by Sir Thomas Willis in 1674, in which he described esophageal dilatation with whalebone in a patient who had dysphagia. Hurst, in 1927, first described the term achalasia, which means failure of relaxation. The first case of achalasia cardia in an infant was reported by King in 1953 [2]. It can be primary (idiopathic) or secondary. Etiologies include viral infections, environmental factors, autoimmunity, and genetic factors. Genes responsible for achalasia are those encoding for receptors of vasoactive intestinal peptide, nitric oxide synthase, aladin, and interleukin-23. It is also associated with trisomy 21, glucocorticoid insufficiency, congenital hypoventilation syndrome, familial dysautonomia, Chagas disease, eosinophilic esophagitis, achalasia alacrimia, and adrenocorticotropic hormone insensitivity syndrome (triple-A syndrome) [3]. Infants usually present with regurgitation of feeds, non-bilious vomiting of uncurdled milk, recurrent chest infections, and failure to thrive. A barium swallow study helps in the preliminary diagnosis of achalasia, but esophageal manometry confirms the diagnosis [4]. Esophageal manometry is technically difficult for infants. Medical management has not been recommended for infants, and surgical management is the mainstay of treatment.

## Case presentation

**Figure 1:**
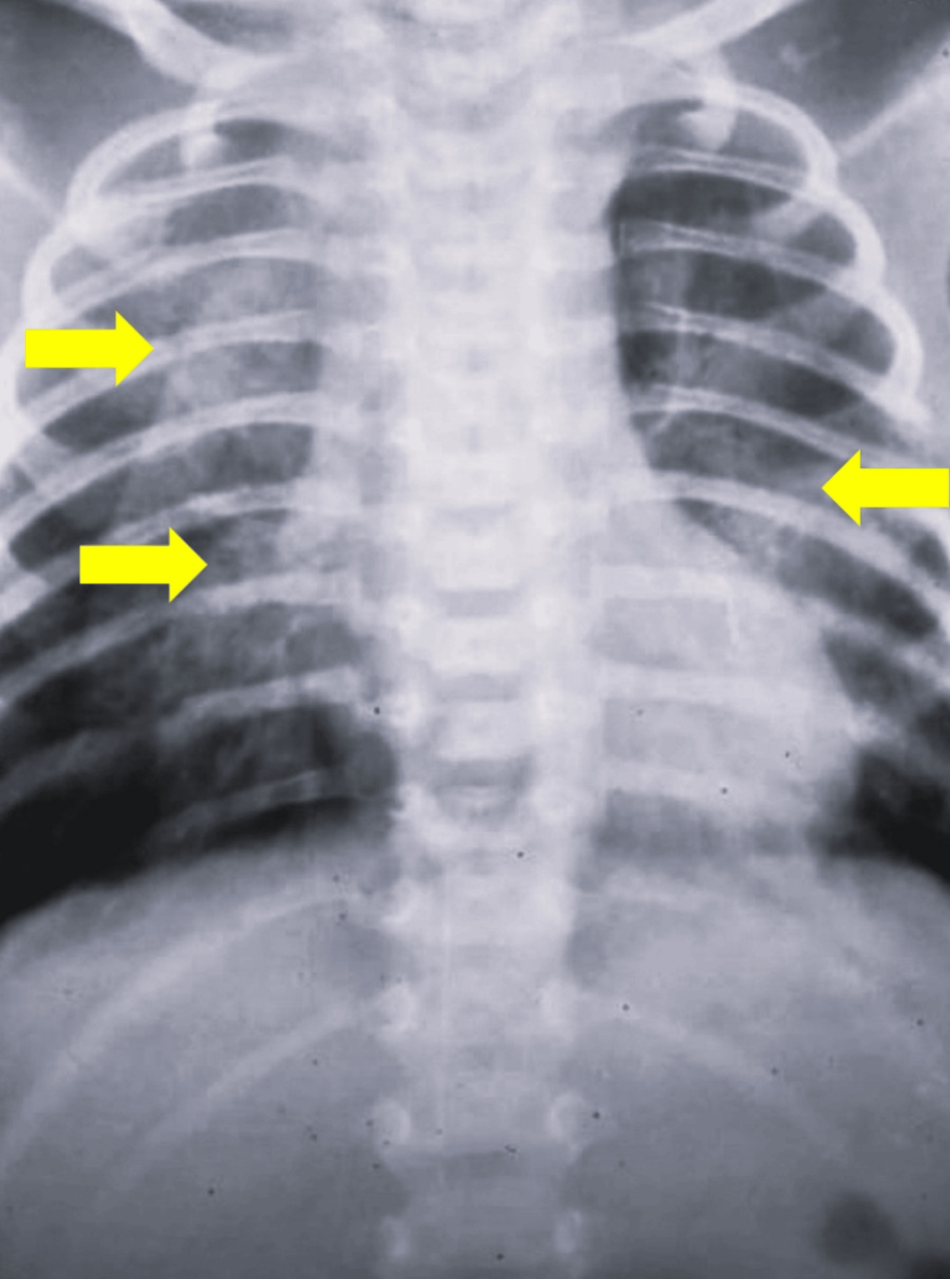
Chest X-ray of the baby. Inhomogenous opacities noted in the apical and posterior segments of the middle lobe of the right lung and the superior segment of the left lung, suggestive of aspiration pneumonia.

A full-term female baby born to third-degree consanguineous parents, fourth in birth order by lower-segment cesarean section, cried immediately after birth with no history of resuscitation. Her birth weight was 2.4 kg. Direct breastfeeding was initiated within one hour of delivery. The baby vomited uncurdled milk immediately after each breastfeeding which was associated with a cough. There was no frothing or drooling of saliva. Hence, the baby was admitted to the neonatal intensive care unit (NICU). She passed urine and meconium within 24 hours of life. In the NICU, the baby was on nasogastric (NG) feeds, and the sepsis screen was negative. Because of neonatal jaundice, the baby received phototherapy for 24 hours. As the baby was tolerating nasogastric feeds well, direct breastfeeds were initiated but could not be tolerated. The baby developed aspiration pneumonia and was kept nil per oral. NG feeds were given after two days and received intravenous (IV) antibiotics for one week. The baby had three hospital admissions in two months for similar complaints. At the age of two months, she was referred to our institute. She was admitted, and further workups were done. Her admission weight was 3 kg (lower than the first centile), length was 53 cm (between the first and third centiles), and head circumference was 36 cm (in the third centile). On examination, the baby was active, pallor was present, there was mild tachypnea, no murmurs, and no obvious congenital anomalies. She was hemodynamically stable, maintaining saturation in room air. Investigations showed hemoglobin of 10 g/dL, elevated white blood cell count of 17,720 cells/mm^3^, and a chest X-ray showed pneumonia, probably due to aspiration (Figure 1). The baby was treated for aspiration pneumonia.

**Figure 2:**
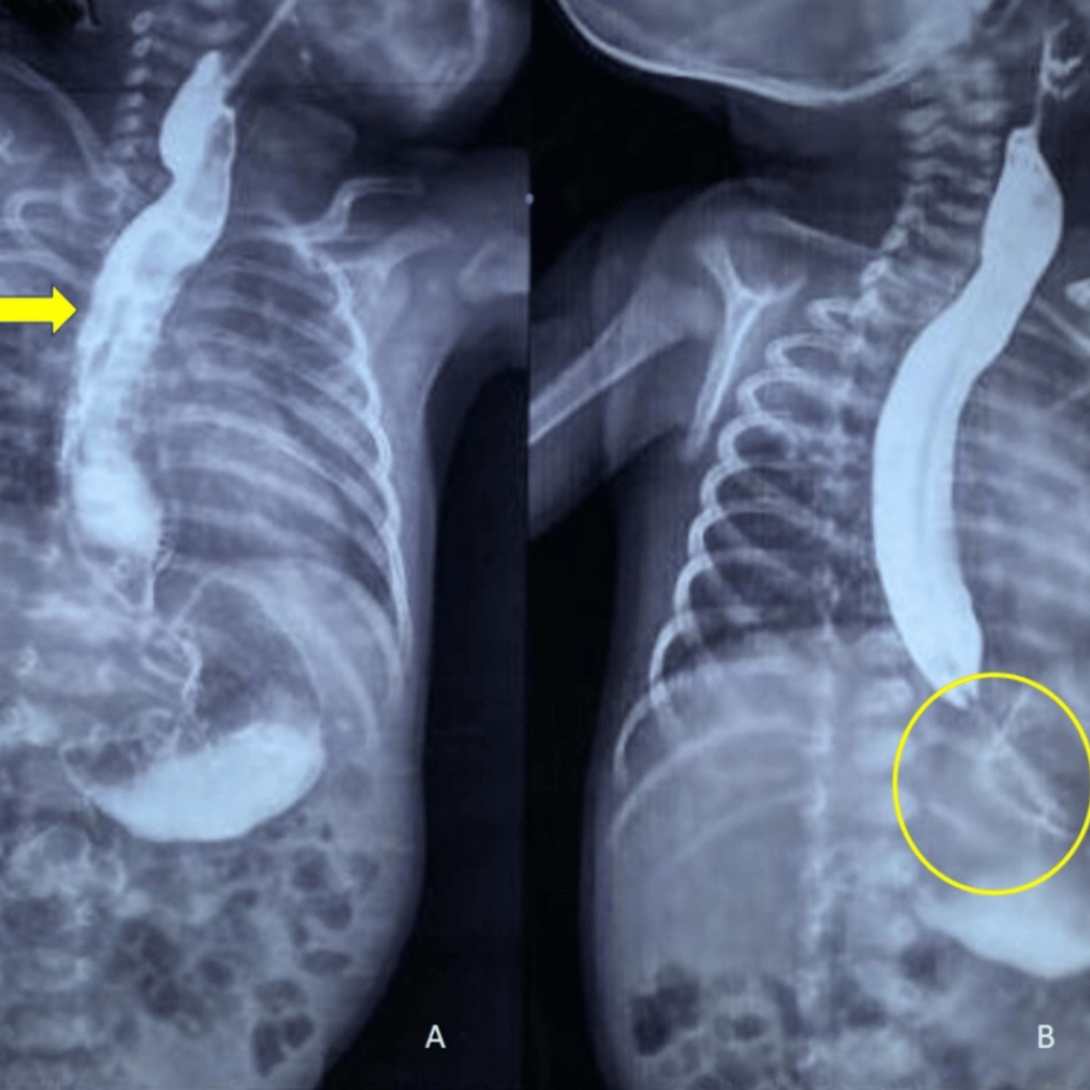
Barium swallow study. A: dilated esophagus; B: narrowing at the lower esophageal sphincter region with a bird beak appearance.

We suspected the following differentials as a possibility: lower esophageal web, stenosis, and H-type tracheoesophageal fistula. A barium swallow study showed a dilated esophagus with narrowing at the LES region with a bird beak appearance, no spillage of dye into the lung fields, no fistulous connection, and no extravasation of dye, which was suggestive of achalasia cardia (Figure 2).

**Figure 3:**
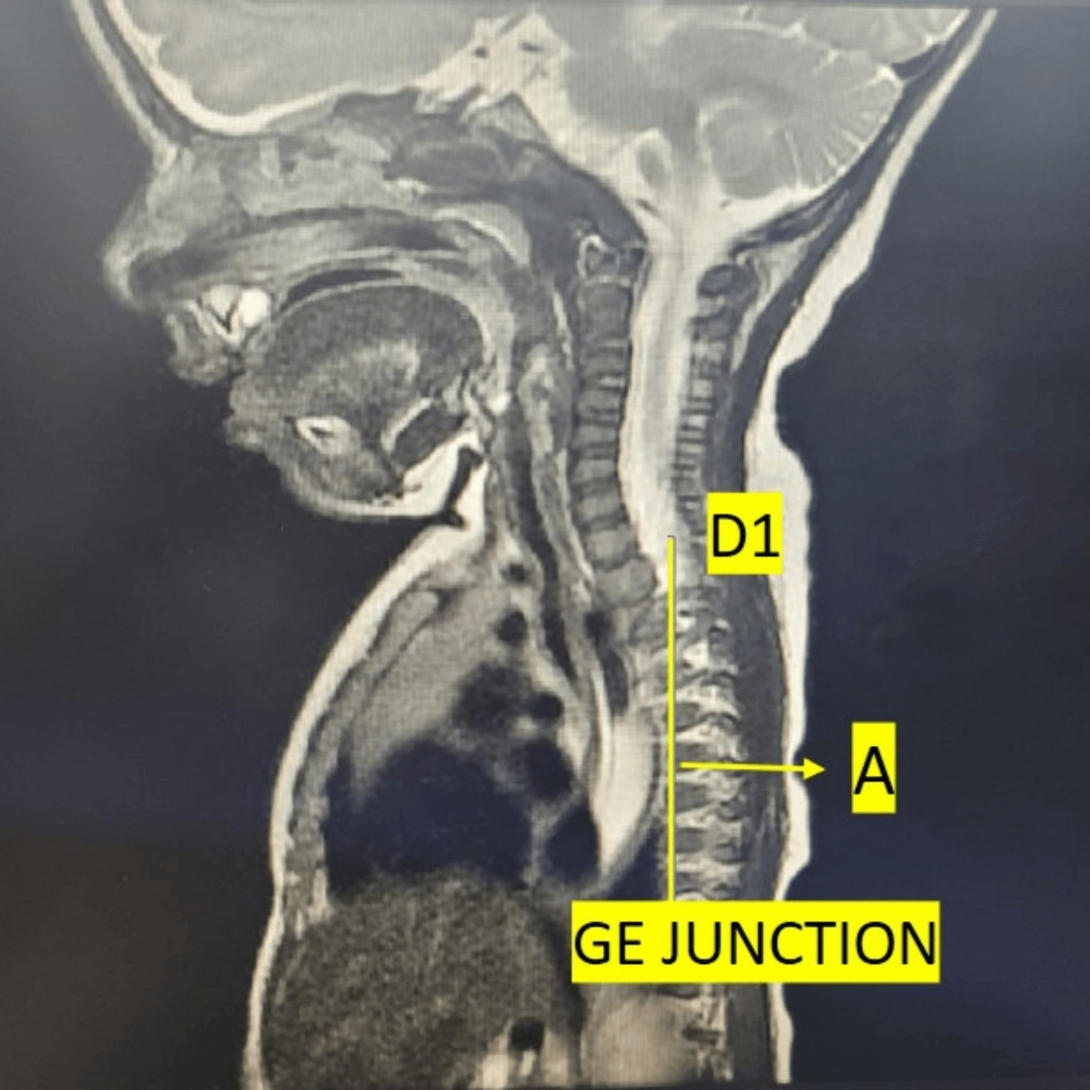
Sagittal T2-weighted MRI of the chest. A: Abnormal dilatation of the entire esophagus from the D1 level to the gastroesophageal (GE) junction with air-fluid levels, suggestive of achalasia cardia; D1: first vertebra of the dorsal spine

We initially started the patient on pharmacological treatment, i.e., a calcium channel blocker. Following a pediatric surgeon referral, surgical management was planned. A medical gastroenterologist’s opinion was taken for endoscopy to rule out any structural anomalies of the esophagus. Because of procedural difficulty, an MRI of the chest was advised for assessing esophageal anatomy. MRI showed abnormal dilatation of the entire esophagus from the D1 level to the gastro-esophageal junction with an air-fluid level and a narrowed lower end of the esophagus, suggestive of achalasia cardia (Figure 3).

Neurosonography, 2D echocardiography, and ultrasonography of the abdomen were normal. After the clinical resolution of aspiration pneumonia, the baby underwent Heller’s esophagomyotomy and Nissen’s fundoplication through the abdominal route by a pediatric surgeon. A blood transfusion was done before surgery given anemia. The postoperative period was uneventful. Initially, the baby was kept nil per oral; later, NG feeds followed by direct oral feeds were initiated, with no further episodes of vomiting. At the time of writing, the wound is healthy, the baby is gaining weight, and is under regular follow-up with a pediatrician and pediatric surgeon.

## Discussion

The reported incidence of achalasia cardia in children is 0.11/100,000, in contrast to adults with an incidence of 1/100,000. Only 3-5% of cases occur in children and fewer than 0.5% in neonates. There is no sex predilection for the disease. The most important symptom in newborns with achalasia cardia is vomiting of uncurdled milk, which may be common in regurgitation due to a faulty feeding technique or overfeeding. Hence, it is easily missed initially. Other features of achalasia cardia, i.e., recurrent aspiration pneumonia, refusal to feed, and failure to thrive, are also seen in gastroesophageal reflux disease (GERD), which is a most common problem in infants [5]. Babies with achalasia cardia may tolerate NG/orogastric tube feeds, like in our case. By definition, an assessment of esophageal motor function is essential in the diagnosis of achalasia. The gold standard of investigation for achalasia is esophageal manometry. The findings include increased basal pressure, abnormal relaxation of the LES, aperistalsis during wet swallows, and an increased gradient across the gastroesophageal junction [6]. Esophageal manometry helps in guiding the length and completeness of the myotomy, and it helps monitor the response to therapy. For any symptoms of recurrence, manometry should be done during follow-up. However, it is not easily available in all the centers [6]. However, manometry requires experience and is technically difficult for newborns or infants [7,8]. A dilated esophagus with smooth tapering in a “bird beak” fashion is seen in the esophageal swallow study. To rule out structural abnormalities such as congenital lower esophageal stenosis or web as well as to delineate esophageal anatomy, an MRI of the chest was done in our case, which also revealed features suggestive of achalasia cardia. Calcium channel blockers are used in adults to treat achalasia. However, there are no studies supporting their use in children [9]. Hence, after discussing with the pediatric surgeon, the plan for definitive surgical management, i.e., Heller’s esophagomyotomy with Nissen’s fundoplication, was made. Peroral endoscopic myotomy is utilized for the treatment of achalasia in adults, but its safety, efficacy, and feasibility are not studied in children [5]. Other modalities of treatment described in the literature with various success rates are local injections of botulinum toxin, which have been tried with some success rates in adults; however, optimal dosing and injection frequency have not been defined in children [1]. Mechanical therapy, which includes esophageal dilatation with pneumatic dilators, has been tried in older children, but the risk of esophageal perforation and recurrence of symptoms within six months are the main concerns [1,10].

## Conclusions

Achalasia cardia in neonates is an exceptionally rare condition. For neonates or infants presenting with symptoms of esophageal obstruction or GERD, particularly with severe failure to thrive and no response to anti-reflux medications, achalasia cardia should be considered as a differential diagnosis. Prompt investigation with a barium swallow study is crucial. Early diagnosis and timely surgical intervention are essential for a successful outcome.
